# Factors Associated With US Adults’ Likelihood of Accepting COVID-19 Vaccination

**DOI:** 10.1001/jamanetworkopen.2020.25594

**Published:** 2020-10-20

**Authors:** Sarah Kreps, Sandip Prasad, John S. Brownstein, Yulin Hswen, Brian T. Garibaldi, Baobao Zhang, Douglas L. Kriner

**Affiliations:** 1 Department of Government, Cornell University, Ithaca, New York; 2 Atlantic Medical Group, Morristown Medical Center, Morris Township, New Jersey; 3 Department of Pediatrics, Harvard Medical School, Boston, Massachusetts; 4 Computational Epidemiology Lab, Boston Children’s Hospital, Boston, Massachusetts; 5 Johns Hopkins Biocontainment Unit, Johns Hopkins University School of Medicine, Baltimore, Maryland

## Abstract

**Importance:**

The development of a coronavirus disease 2019 (COVID-19) vaccine has progressed at unprecedented speed. Widespread public uptake of the vaccine is crucial to stem the pandemic.

**Objective:**

To examine the factors associated with survey participants’ self-reported likelihood of selecting and receiving a hypothetical COVID-19 vaccine.

**Design, Setting, and Participants:**

A survey study of a nonprobability convenience sample of 2000 recruited participants including a choice-based conjoint analysis was conducted to estimate respondents’ probability of choosing a vaccine and willingness to receive vaccination. Participants were asked to evaluate their willingness to receive each hypothetical vaccine individually. The survey presented respondents with 5 choice tasks. In each, participants evaluated 2 hypothetical COVID-19 vaccines and were asked whether they would choose vaccine A, vaccine B, or neither vaccine. Vaccine attributes included efficacy, protection duration, major adverse effects, minor adverse effects, US Food and Drug Administration (FDA) approval process, national origin of vaccine, and endorsement. Levels of each attribute for each vaccine were randomly assigned, and attribute order was randomized across participants. Survey data were collected on July 9, 2020.

**Main Outcomes and Measures:**

Average marginal component effect sizes and marginal means were calculated to estimate the relationship between each vaccine attribute level and the probability of the respondent choosing a vaccine and self-reported willingness to receive vaccination.

**Results:**

A total of 1971 US adults responded to the survey (median age, 43 [interquartile range, 30-58] years); 999 (51%) were women, 1432 (73%) White, 277 (14%) were Black, and 190 (10%) were Latinx. An increase in efficacy from 50% to 70% was associated with a higher probability of choosing a vaccine (coefficient, 0.07; 95% CI, 0.06-0.09), and an increase from 50% to 90% was associated with a higher probability of choosing a vaccine (coefficient, 0.16; 95% CI, 0.15-0.18). An increase in protection duration from 1 to 5 years was associated with a higher probability of choosing a vaccine (coefficient, 0.05 95% CI, 0.04-0.07). A decrease in the incidence of major adverse effects from 1 in 10 000 to 1 in 1 000 000 was associated with a higher probability of choosing a vaccine (coefficient, 0.07; 95% CI, 0.05-0.08). An FDA emergency use authorization was associated with a lower probability of choosing a vaccine (coefficient, −0.03; 95% CI, −0.04 to −0.01) compared with full FDA approval. A vaccine that originated from a non-US country was associated with a lower probability of choosing a vaccine (China: −0.13 [95% CI, −0.15 to −0.11]; UK: −0.04 [95% CI, −0.06 to −0.02]). Endorsements from the US Centers for Disease Control and Prevention (coefficient, 0.09; 95% CI, 0.07-0.11) and the World Health Organization (coefficient, 0.06; 95% CI, 0.04-0.08), compared with an endorsement from President Trump were associated with higher probabilities of choosing a vaccine. Analyses of participants’ willingness to receive each vaccine when assessed individually yielded similar results. An increase in efficacy from 50% to 90% was associated with a 10% higher marginal mean willingness to receive a vaccine (from 0.51 to 0.61). A reduction in the incidence of major side effects was associated with a 4% higher marginal mean willingness to receive a vaccine (from 0.54 to 0.58). A vaccine originating in China was associated with a 10% lower willingness to receive a vaccine vs one developed in the US (from 0.60 to 0.50) Endorsements from the Centers for Disease Control and Prevention and World Health Organization were associated with increases in willingness to receive a vaccine (7% and 6%, respectively) from a baseline endorsement by President Trump (from 0.52 to 0.59 and from 0.52 to 0.58, respectively).

**Conclusions and Relevance:**

In this survey study of US adults, vaccine-related attributes and political characteristics were associated with self-reported preferences for choosing a hypothetical COVID-19 vaccine and self-reported willingness to receive vaccination. These results may help inform public health campaigns to address vaccine hesitancy when a COVID-19 vaccine becomes available.

## Key Points

**Question:**

What factors are associated with US adults’ choice of and willingness to accept a hypothetical COVID-19 vaccine?

**Findings:**

In this survey study of a national sample of 1971 US adults, vaccine-related attributes (eg, vaccine efficacy, adverse effects, and protection duration) and political factors (eg, US Food and Drug Administration approval process, national origin of vaccine, and endorsements) were associated with preferences for choosing a hypothetical COVID-19 vaccine. Health care attitudes and practices, political partisanship, and demographic characteristics, including age, sex, and race/ethnicity, were also associated with willingness to receive a vaccination.

**Meaning:**

The results of this survey study may help inform public health campaigns to address vaccine hesitancy.

## Teaser

This survey study evaluates the factors associated with US adults’ choice of and willingness to accept a hypothetical COVID-19 vaccine.

## Introduction

In April 2020, the United States established Operation Warp Speed to develop a coronavirus disease 2019 (COVID-19) vaccine and produce 300 million doses by January 2021. History suggests that the initiative is aptly named. As Barney S. Graham notes, “Vaccine development is usually measured in decades, so having access to approved vaccines available for large-scale distribution before the end of 2020 or even 2021 would be unprecedented.”^1^

Timeliness of vaccine development and availability are not the only obstacles from a public health standpoint. Once a vaccine is developed, a sufficient share of the population must be vaccinated to reach herd immunity and prevent wider spread in the community. A growing antivaccination movement threatens such efforts in the United States,^2^ Europe,^3^ and Asia.^4^

Existing surveys suggest that US public support for a hypothetical COVID-19 vaccination may not reach the thresholds necessary to achieve herd immunity, which is estimated at 70% for COVID-19.^5^ In a survey of 1056 US adults, 49% said they would take a vaccine once available, 31% were unsure, and 20% said they would not be vaccinated.^6^ In other polls, 71% of US adults reported that they would be vaccinated.^7^ Although they are useful first steps in gauging potential vaccine acceptance, these surveys solicit responses to a generic vaccine and do not account for how its potential attributes, such as efficacy, location of vaccine development, and risk of adverse effects, affect public acceptance.

Understanding how different vaccine attributes affect individual preferences about vaccination may help inform public health authorities about what types of endorsements, incentives, or messages are necessary to achieve broader community uptake. In this survey study, we deploy a choice-based conjoint analysis that included 7 attributes of vaccine candidates to understand which factors were associated with the choice of a vaccine and self-reported willingness to receive COVID-19 vaccination.

## Methods

For this survey study, we conducted an online survey on July 9, 2020, to assess individual COVID-19 vaccine preferences. Cornell University’s Institutional Review Board for Human Participant Research approved the study, and the study’s pre-analysis plan was registered through the Open Science Framework.^8^ After providing informed consent on the initial screen of the online survey, participants were presented with a choice-based conjoint experiment to identify factors associated with self-reported likelihood of vaccination. This study followed the American Association for Public Opinion Research (AAPOR) reporting guideline.

### Conjoint Analysis Methodology

Conjoint analysis is a survey-based method from market research^9^ that has been used in health research,^10^ including research examining factors associated with public preferences concerning vaccines. The approach approximates actual decisions and has been shown to moderately inform the estimation of future health behaviors.^11^

### Survey

In this study, respondents were informed about hypothetical COVID-19 vaccines under development. In the presented vaccine profiles, 7 categories of vaccine attributes were varied, drawing on attributes found to be important in previous vaccine acceptance studies as well as early evidence about promising COVID-19 vaccine candidates. Four of the attributes—efficacy,^12,13,14,15^ the incidence of major^16,17^ and minor adverse effects,^18,19^ and protection duration^17,18^are commonly analyzed in the public vaccine acceptance literature. Attribute levels were drawn from both prior research and emerging speculation about the likely characteristics of a COVID-19 vaccine, for example, that its efficacy could be lower and protection duration shorter, like the flu vaccine, than other vaccines, such as for measles, mumps, and rubella or polio.^20^ Three additional attributes—whether the vaccine received full US Food and Drug Administration (FDA) approval or an emergency use authorization (EUA),^21,22^ the nation of origin of the vaccine, and the agency or political leader who endorsed the vaccine^12,23,24^—were used to examine political features of a COVID-19 vaccine. Table 1 presents each attribute and level examined in the experiment. The choice-based conjoint analysis can examine the potential influence of an extensive set of vaccine features on individual preferences in ways that would be difficult with a standard survey experimental design.

**Table 1.** Attributes and Attribute Levels for Hypothetical COVID-19 Vaccines

Vaccine attribute	Level
Efficacy (protection against severe symptoms)	50%
	70%
	90%
Protection duration	1 y
	5 y
Risk of severe side effects (hospitalization or death)	1 in 10 000
	1 in 1 000 000
Risk of mild side effects (flu-like symptoms)	1 in 10
	1 in 30
Government authorization	FDA emergency use authorization^a^
	FDA approved and licensed^b^
Vaccine origin	United States
	China
	United Kingdom
Recommended by	Trump
	Biden
	Centers for Disease Control and Prevention
	World Health Organization

Abbreviation: COVID-19, coronavirus disease 2019.

^a^ The vaccine has received an emergency use authorization from the US Food and Drug Administration (FDA), which allows the expedited use of promising drugs that the FDA reasonably believes may be effective in combatting the virus.

^b^ The vaccine has been approved and licensed by the US FDA.

The survey presented respondents with 5 choice tasks. In each task, participants evaluated 2 hypothetical COVID-19 vaccines and were asked whether they would choose vaccine A, vaccine B, or neither vaccine. Levels of each attribute for each vaccine were randomly assigned, and attribute order was randomized across participants. A sample choice set is presented in eFigure 1 in the Supplement. After making a discrete choice between the 2 vaccines, respondents were also asked to indicate how likely or unlikely they would be to receive each vaccine individually on a 7-point Likert scale from extremely unlikely to extremely likely. The 7-point Likert scale is a standard measurement tool. Before launching the survey on the full sample, a pilot was conducted on 50 participants to ensure that our prompts and questions were not confusing participants. The respondents in the pilot sample did not express confusion or concern with any design elements.

### Study Population and Data Collection

The following demographic characteristics of participants were collected to assess the representativeness of our sample: age, sex, race/ethnicity, education, income level, and political partisanship and ideology. To determine the survey sample size, the method outlined by Kelley and Maxwell^25^ was used for ensuring sufficient precision in coefficient estimates. Given the study parameters, a sample size of 1600 was determined sufficient to produce confidence intervals 80% of the time for each standardized regression coefficient of less than plus or minus 0.05. To be conservative, a target sample size of 2000 was chosen—a considerably larger sample than many other health choice studies.^26^ The largest number of treatment groups per attribute was 4. For comparisons between 2 treatment groups, this sample size allowed detection of treatment effect sizes of 0.2 standard deviations (a small effect size), with a type I error rate of α = .05 and a power of 0.89.

On July 9, 2020, 3708 US adults were contacted and 2000 recruited to take a 15-minute survey through the Lucid platform. This platform employs quota-based sampling to approximate nationally representative samples in terms of demographic characteristics. All participants were recruited indirectly through the firm Lucid. Lucid charged a set fee for providing the agreed-upon number of survey participants, which was paid directly to Lucid. The authors had no influence over the terms of compensation agreed on between Lucid and those who accepted their invitation to take our survey. The 2013 AAPOR report on nonprobability samples recommends using probability samples for descriptive statistics for populations but finds that nonprobability samples are acceptable for studying relationships between variables, such as when conducting an experiment.^27^ Thus, estimates of population values are not reported,^28^ but rather, estimated effect sizes were the focus of the analyses for the association between randomly assigned vaccine attributes and expressed willingness to receive the vaccine among the sample. Recent research has shown that randomized experimental effect sizes estimated on this platform’s nonprobability samples approximate those found using national probability surveys.^29^

### Statistical Analysis

A fully randomized conjoint is a full factorial design; the number of attributes and levels in the current study design yields 576 unique profiles (4^1^ × 3^2^ × 2^4^), in which all possible combinations may not be observed. However, randomization assures that attributes are orthogonal, which allows the estimation of the marginal contribution of each attribute. To analyze responses to the discrete choice question, the methods described by Hainmueller and colleagues^30^ were used with an ordinary least squares (OLS) regression, with standard errors clustered on respondent to estimate the average marginal component effect sizes for each attribute. For each regression coefficient, 95% CIs were calculated from these clustered, heteroskedasticity-robust standard errors. The average marginal component effect sizes represent the mean difference in a respondent choosing a vaccine when comparing 2 different attribute values—for example, 50% efficacy vs 90% efficacy—averaged across all possible combinations of the other vaccine attribute values. The average marginal component effect sizes were nonparametrically identified under a modest set of assumptions, many of which (such as randomization of attribute levels) were determined by design.

To analyze responses to follow-up questions asking participants to evaluate each vaccine individually, a benchmark OLS regression was estimated, and marginal means, which measure the level of favorability toward a vaccine for each attribute-level averaging across all other features, were calculated.^31^ To assess the associations between underlying health characteristics, general medical preferences, demographic characteristics, and willingness to receive a COVID-19 vaccine, both benchmark OLS regressions were also estimated with a series of control variables, including self-reported demographic characteristics. This method focused on results from the model analyzing self-reported willingness to receive each vaccine individually, as these results were the most immediately interpretable substantively. We reported any result with a 2-tailed *P* < .05 as statistically significant. As a robustness check, we also identified coefficients with *P* values below the adjusted target *P* value (adjusting for α = .05) calculated via the Benjamini-Hochberg correction and controlling for the false discovery rate in multiple comparisons. All statistical analyses were conducted using Stata, version 15 (StataCorp LLC).

## Results

On July 9, 2020, Lucid contacted 3708 US adults to recruit a convenience sample of 2000 participants who consented to begin the survey. From this sample, 1971 completed the full questionnaire. Table 2 presents sample demographic characteristics, which largely reflect those of the US population as a whole. A total of 999 participants (51%) were women; 1432 (73%) were White, 277 (14%) were Black, and 190 (10%) were Latinx; the median age was 43 (interquartile range, 30-58) years. Comparisons of the sample demographic characteristics to those of other prominent social science surveys and US Census figures are shown in eTable 1 in the Supplement.

**Table 2.** Demographic Characteristics

Characteristic	No. (%)
Age, y	
18-29	456 (23)
30-44	577 (29)
45-59	490 (25)
≥60	448 (23)
Sex	
Male	972 (49)
Female	999 (51)
Race/ethnicity^a^	
White	1432 (73)
Black	277 (14)
Latinx	190 (10)
Asian	117 (6)
Native American	45 (2)
Other	39 (2)
Educational attainment	
Less than high school	34 (2)
High school/GED	410 (21)
Some college	583 (30)
4-y College degree	469 (24)
Graduate school	475 (24)
Income	
<$20 000	328 (17)
$20 000-$39 999	376 (19)
$40 000-$59 999	347 (18)
$60 000-$79 999	277 (14)
$80 000-$99 999	184 (9)
≥$100 000	459 (23)
Political partisanship	
Democrat (including those who leaned toward the party)	917 (47)
Republican (including those who leaned toward the party)	857 (43)
Political ideology	
Very liberal	258 (13)
Liberal	286 (15)
Somewhat liberal	152 (8)
Moderate	660 (33)
Somewhat conservative	178 (9)
Conservative	211 (11)
Very conservative	226 (11)

Abbreviation: GED, General Educational Development.

^a^ Survey allowed for multiple options to be selected.

Across all choice sets, in 7784 of 9855 cases (79%), participants chose to take 1 of the 2 hypothetical vaccines presented in the discrete choice question; in 2071 of 9855 cases (21%), participants chose neither. When asked to evaluate vaccines individually across all hypothetical profiles (19 710), in 11 058 cases (56%), participants said they would be extremely, moderately, or slightly likely to choose the presented vaccine.

**Figure 1.**
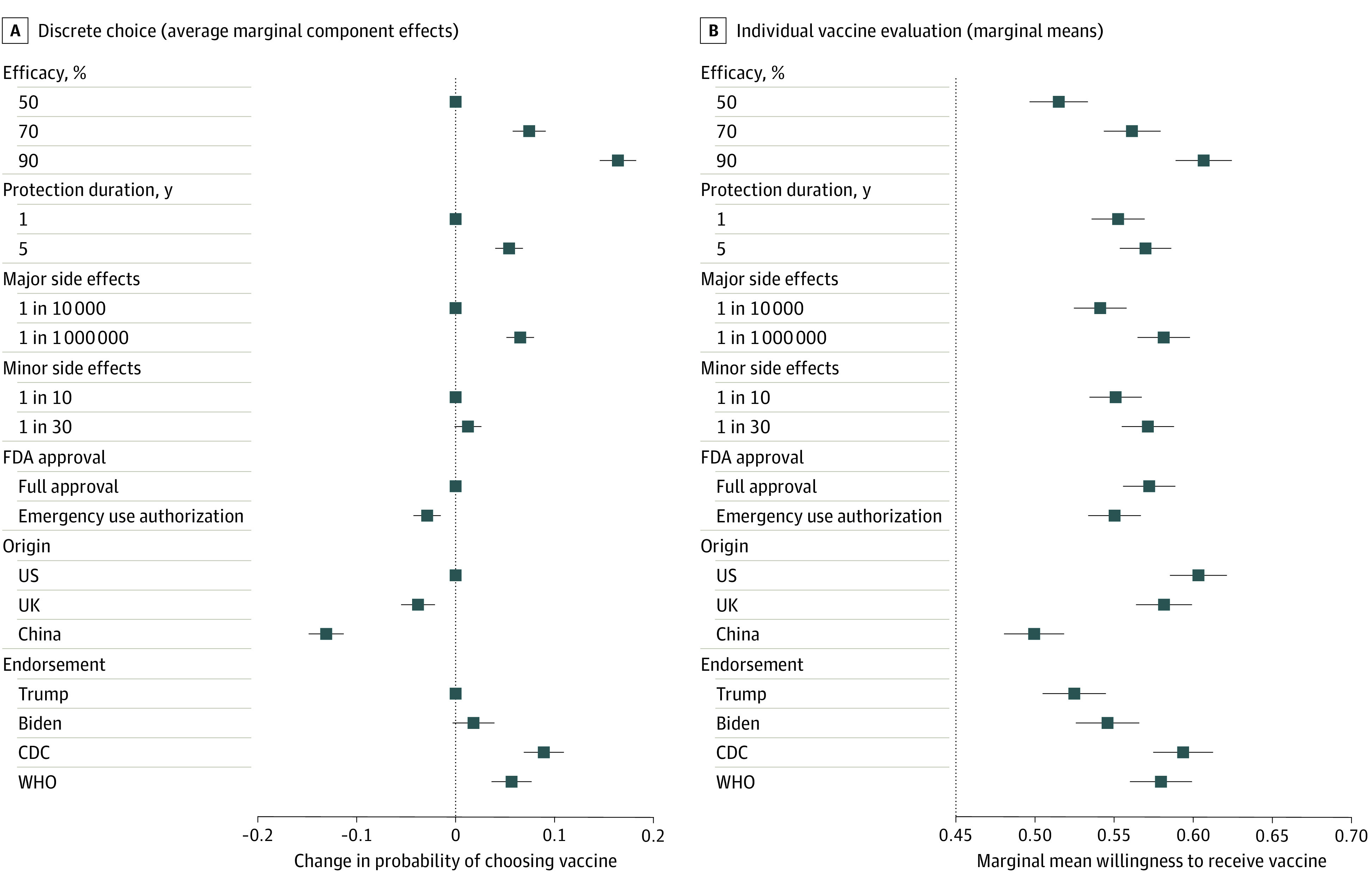
Vaccine Attributes and Vaccination Preferences Panel A shows the estimated effect size of each attribute value on the probability of a study respondent choosing a hypothetical coronavirus disease 2019 (COVID-19) vaccine. The average marginal component effect sizes plotted here are the regression coefficients reported in Model 1 in Table 3. The points without error bars denote the base level value for each attribute. Panel B shows the marginal means for each attribute value for willingness to receive a hypothetical COVID-19 vaccine. Estimates are based on Model 3 in Table 3, and numerical point estimates and confidence intervals are reported in eTable 2 in the Supplement. In both panels, error bars represent 95% CIs around each point estimate. CDC indicates Centers for Disease Control and Prevention; FDA, US Food and Drug Administration; and WHO, World Health Organization.

Model 1 of Table 3 presents a benchmark OLS regression estimating the effect sizes of each attribute level on the probability of choosing a vaccine in the discrete choice question. Model 3 of Table 3 presents a similar OLS benchmark model for self-reported willingness to receive each vaccine individually. The top panel of Figure 1 presents the estimated effect sizes for each vaccine attribute level on the probability of choosing a hypothetical COVID-19 vaccine from the corresponding baseline level of that attribute (from Model 1 of Table 3). The bottom panel of Figure 1 presents marginal means from Model 3 of Table 3. This approach offers information about vaccination likelihood at all feature levels. For point estimates and confidence intervals, see eTable 2 in the Supplement. Additional analyses presenting marginal means for the discrete choice question and average marginal component effect sizes for the individual vaccine evaluations are reported in eFigure 2 in the Supplement.

**Table 3.** Attributes and Vaccine Preferences^a^

Attribute	Discrete choice question				Individual vaccine evaluation			
	Model 1		Model 2		Model 3		Model 4	
	Coefficient (95% CI)	*P* value	Coefficient (95% CI)	*P* value	Coefficient (95% CI)	*P* value	Coefficient (95% CI)	*P* value
Efficacy: 70%	0.07 (0.06 to 0.09)	<.001^b^	0.08 (0.06 to 0.09)	<.001^b^	0.05 (0.03 to 0.06)	<.001^b^	0.05 (0.03 to 0.07)	<.001^b^
Efficacy: 90%	0.16 (0.15 to 0.18)	<.001^b^	0.17 (0.15 to 0.18)	<.001^b^	0.09 (0.07 to 0.11)	<.001^b^	0.10 (0.08 to 0.11)	<.001^b^
Duration: 5 y	0.05 (0.04 to 0.07)	<.001^b^	0.05 (0.04 to 0.07)	<.001^b^	0.02 (0.003 to 0.03)	.01^b^	0.01 (0.001 to 0.03)	.03^b^
Major adverse effects: 1 in 1 000 000	0.07 (0.05 to 0.08)	<.001^b^	0.07 (0.05 to 0.08)	<.001^b^	0.04 (0.03 to 0.05)	<.001^b^	0.04 (0.03 to 0.06)	<.001^b^
Minor adverse effects: 1 in 30	0.01 (−0.001 to 0.03)	0.07	0.01 (0.00 to 0.03)	.04	0.02 (0.01 to 0.03)	.004^b^	0.02 (0.01 to 0.04)	.001^b^
FDA: EUA	−0.03 (−0.04 to −0.01)	<.001^b^	−0.03 (−0.04 to −0.02)	<.001^b^	−0.02 (−0.04 to −0.01)	.003^b^	−0.02 (−0.04 to −0.01)	.001^b^
Origin: United Kingdom	−0.04 (−0.06 to −0.02)	<.001^b^	−0.04 (−0.05 to −0.02)	<.001^b^	−0.02 (−0.04 to −0.005)	.01^b^	−0.02 (−0.04 to −0.004)	.01^b^
Origin: China	−0.13 (−0.15 to −0.11)	<.001^b^	−0.13 (−0.15 to −0.11)	<.001^b^	−0.10 (−0.12 to −0.08)	<.001^b^	−0.10 (−0.12 to −0.09)	<.001^b^
Endorsed: Biden	0.02 (−0.003 to 0.04)	.09	0.02 (−0.004 to 0.04)	.11	0.02 (−0.001 to 0.04)	.06	0.02 (−0.002 to 0.04)	.08
Endorsed: CDC	0.09 (0.07 to 0.11)	<.001^b^	0.09 (0.07 to 0.11)	<.001^b^	0.07 (0.05 to 0.09)	<.001^b^	0.07 (0.05 to 0.09)	<.001^b^
Endorsed: WHO	0.06 (0.04 to 0.08)	<.001^b^	0.06 (0.04 to 0.08)	<.001^b^	0.05 (0.03 to 0.08)	<.001^b^	0.06 (0.03 to 0.08)	<.001^b^
Democrat	NA	NA	0.06 (0.04 to 0.08)	<.001^b^	NA	NA	0.12 (0.08 to 0.16)	<.001^b^
Republican	NA	NA	0.03 (0.01 to 0.05)	.009^b^	NA	NA	0.05 (0.01 to 0.09)	.01^b^
Female	NA	NA	−0.02 (−0.04 to −0.01)	.001^b^	NA	NA	−0.08 (−0.11 to −0.05)	<.001^b^
Age (in decades)	NA	NA	−0.004 (−0.009 to 0.001)	.09	NA	NA	−0.02 (−0.02 to −0.01)	<.001^b^
Education	NA	NA	0.01 (0.01 to 0.02)	<.001^b^	NA	NA	0.03 (0.02 to 0.04)	<.001^b^
Flu vaccination frequency	NA	NA	0.03 (0.02 to 0.03)	<.001^b^	NA	NA	0.04 (0.03 to 0.05)	<.001^b^
Uninsured	NA	NA	−0.03 (−0.05 to −0.02)	<.001^b^	NA	NA	−0.06 (−0.08 to −0.04)	<.001^b^
Pharma favorability	NA	NA	0.03 (0.02 to 0.04)	<.001^b^	NA	NA	0.08 (0.07 to 0.09)	<.001^b^
Know a COVID-19 case	NA	NA	0.02 (0.004 to 0.03)	.01^b^	NA	NA	0.05 (0.02 to 0.08)	<.001^b^
Worst of pandemic to come	NA	NA	0.04 (0.02 to 0.05)	<.001^b^	NA	NA	0.04 (0.01 to 0.06)	.01^b^
Non-evangelical Christian	NA	NA	0.02 (−0.02 to 0.05)	.34	NA	NA	−0.01 (−0.08 to 0.05)	.65
Evangelical Christian	NA	NA	0.002 (−0.02 to 0.02)	.81	NA	NA	0.01 (−0.02 to 0.05)	.42
Not religious	NA	NA	0.01 (−0.01 to 0.03)	.45	NA	NA	0.01 (−0.03 to 0.04)	.74
Black	NA	NA	−0.03 (−0.05 to −0.01)	.01^b^	NA	NA	−0.10 (−0.14 to −0.06)	<.001^b^
Latinx	NA	NA	−0.01 (−0.04 to 0.01)	.32	NA	NA	−0.04 (−0.09 to 0.002)	.06
Constant	0.28 (0.25 to 0.30)	<.001	0.01 (−0.04 to 0.07)	.62	0.49 (0.46 to 0.52)	<.001	−0.01 (−0.09 to 0.08)	.88
Observations, No.	19 710	NA	19 710	NA	19 710	NA	19 710	NA

Abbreviations: CDC, Centers for Disease Control and Prevention; COVID-19, coronavirus disease 2019; EUA, emergency use authorization; FDA, US Food and Drug Administration; NA, not applicable; WHO, World Health Organization.

^a^ Data presented as regression coefficients and 95% CIs. Regression coefficients for the randomized vaccine attributes are the average marginal component effects of each attribute level. Models 1 and 2 present results for the discrete choice question, in which subjects chose vaccine A, vaccine B, or neither. Models 3 and 4 present results for subjects’ reported likelihood of taking each vaccine when evaluated individually (coded 1 for slightly, moderately, or extremely likely and 0 if not). Models 1 and 3 are benchmark ordinary least squares regressions used to compute average marginal component effects and marginal means presented graphically in Figure 2.

^b^ *P* values for coefficients are below the adjusted target *P* value (adjusting for α = .05) calculated via the Benjamini-Hochberg correction and controlling for the false discovery rate in multiple comparisons.

When choosing between a pair of hypothetical vaccines, the most important attribute for respondent choice was efficacy. An increase in vaccine efficacy from 50% to 70% was associated with an increase in the probability of choosing a vaccine (coefficient, 0.07; 95% CI, 0.06-0.09), and an increase from 50% to 90% efficacy was associated with an increase in the probability of choosing a vaccine (coefficient, 0.16; 95% CI, 0.15-0.18). A longer protection duration (5 years vs 1 year) was associated with a higher probability of choosing a vaccine (coefficient, 0.05; 95% CI, 0.04-0.07), and a significantly lower incidence of major side effects was associated with a higher probability of choosing the vaccine (coefficient, 0.07; 95% CI, 0.05-0.08).

Political attributes were also associated with vaccine choice. An FDA EUA was associated with a lower probability of choosing a vaccine (coefficient, −0.03; 95% CI, −0.04 to −0.01) compared with a full FDA approval. Respondents were less likely to choose vaccines developed outside of the United States, particularly from China, which was associated with a lower probability of choosing the vaccine (coefficient, −0.13; 95% CI, −0.15 to −0.11). The probability of choosing a vaccine was lowest when the vaccine was recommended by President Trump (the baseline category), although the probability was not significantly higher when the vaccine was recommended by former Vice President Biden (coefficient, 0.02; 95% CI, −0.003 to 0.04). Compared with an endorsement from President Trump, Centers for Disease Control and Prevention and World Health Organization endorsements were associated with higher probabilities of choosing the vaccine (coefficient, 0.09 [95% CI, 0.07-0.11] vs 0.06 [95% CI, 0.04-0.08]).

The marginal means in the bottom panel of Figure 1 display similar findings. Vaccine efficacy was the most important characteristic associated with self-reported willingness to receive a vaccine. An increase in the efficacy of a vaccine from 50% to 90% was associated with an increase in the marginal mean willingness to receive the vaccine of 10% from 0.51 to 0.61. A longer protection duration (5 years vs 1 year) was associated with an increase in the marginal mean willingness to receive a vaccine from 0.55 to 0.57. A decrease in the incidence of major adverse effects, such as hospitalization or death, was associated with a 4% increase in the marginal mean willingness to receive the vaccine from 0.54 to 0.58, while a decrease in the incidence of minor adverse effects was associated with an increase in the marginal mean willingness to receive vaccination from 0.55 to 0.57. An FDA EUA was associated with a decrease in the marginal mean willingness to receive a vaccine from 0.57 under full FDA approval to 0.55. For a vaccine developed in the United States, the marginal mean willingness to receive a vaccine was 0.60. This likelihood decreased slightly to 0.58 for a vaccine developed in the United Kingdom and decreased to 0.50 for a vaccine developed in China. The marginal mean willingness to receive a vaccine was lowest when the vaccine was recommended by President Trump (0.52). The marginal mean willingness to receive a vaccine was slightly higher when the vaccine was recommended by former Vice President Biden (0.55) and significantly higher given a Centers for Disease Control and Prevention (0.59) or World Health Organization (0.58) endorsement. Additional analyses using alternate operationalizations of vaccine acceptance yielded consistent results (eAppendix and eTable 3 in the Supplement).

**Figure 2.**
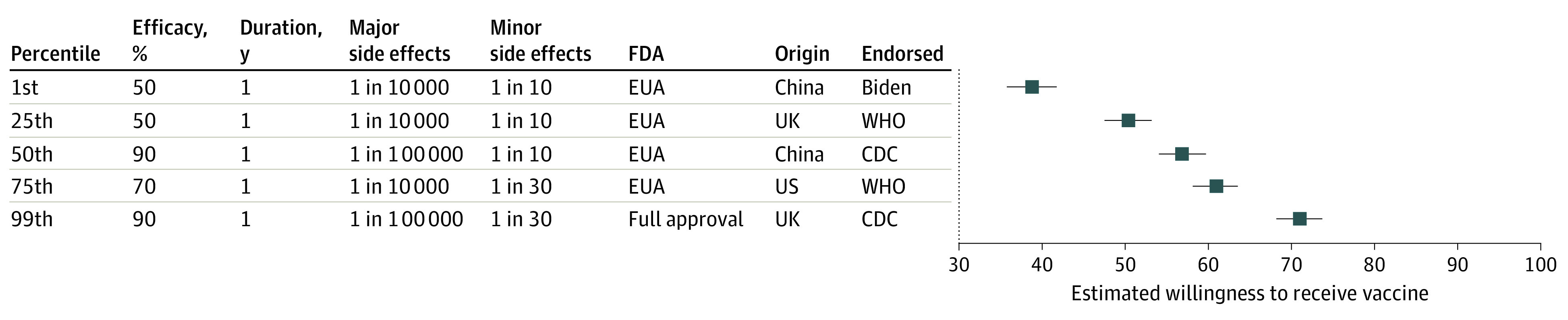
Estimated Willingness to Receive 5 Hypothetical Vaccines This plot shows US adults’ estimated willingness to receive 5 different vaccines. The hypothetical vaccines have the attribute profiles of experimental vaccines at the 1st, 25th, 50th, 75th, and 99th percentiles in terms of estimated willingness to receive vaccination. The estimates are derived from an ordinary least squares regression in which the dependent variable was coded 1 if the individual was at least slightly likely to accept the described vaccine (Model 3 in Table 3). Error bars represent 95% CIs obtained from simulations. CDC indicates Centers for Disease Control and Prevention; EUA, emergency use authorization; FDA, US Food and Drug Administration; and WHO, World Health Organization.

Figure 2 presents a respondent’s estimated willingness to be vaccinated under 5 hypothetical vaccine profiles, which represent the 1st, 25th, 50th, 75th, and 99th percentiles in terms of estimated willingness to receive vaccination. For the first hypothetical vaccine (50% efficacy, a 1-year protection duration, was approved under an FDA EUA, and developed in China), the estimated willingness to receive vaccination was 40% (95% CI, 37%-42%). In the 25th percentile vaccine, which had similar characteristics but was developed in the United Kingdom and endorsed by the World Health Organization, the estimated willingness to receive vaccination was 51% (95% CI, 48%-53%). The median vaccine profile (50th percentile) had an estimated willingness to receive the vaccine of 56% (95% CI, 53%-60%). The 99th percentile vaccine (90% efficacy, 5-year protection duration, low levels of major and minor side effects, full FDA approval, developed in the United Kingdom, and endorsed by the Centers for Disease Control and Prevention), had an estimated willingness to receive vaccination of 71% (95% CI, 68%-74%).

### Additional Correlates of Vaccine Acceptance

In Table 3, demographic control variables in models 2 and 4 examine the associations between underlying health characteristics, general medical preferences, demographics, and vaccination preferences. Results from model 4 are presented herein. Results using the discrete choice dependent variable are substantively similar. Respondents who indicated Democratic political partisanship (coefficient, 0.12; 95% CI, 0.08-0.16) were significantly more likely to report willingness to receive vaccination than those who indicated Republican political partisanship (coefficient, 0.05; 95% CI, 0.01-0.09) or who indicated independent political partisanship (the omitted baseline). In models 2 and 4, Wald tests confirmed that the positive coefficient for the Democratic indicator was numerically larger than that for the Republican indicator (Table 3). Women (coefficient, −0.08; 95% CI, −0.11 to −0.05) and Black respondents (coefficient, −0.10; 95% CI, −0.14 to −0.06) were less likely to indicate willingness to receive a vaccine. Educational attainment (coefficient, 0.03; 95% CI, 0.02-0.04) was associated with a greater willingness to receive vaccination, whereas age (coefficient, −0.02; 95% CI, −0.02 to −0.01) was associated with a lower willingness to receive vaccination. Increased frequency of flu vaccination (coefficient, 0.04; 95% CI, 0.03-0.05) was associated with a greater willingness to receive COVID-19 vaccination, as were more favorable attitudes toward the pharmaceutical industry (coefficient, 0.08; 95% CI, 0.07-0.09). Uninsured adults had a lower willingness to receive a vaccine (coefficient, −0.06; 95% CI, −0.08 to −0.04) than those who had health insurance. Both personal contact with someone diagnosed with COVID-19 (coefficient, 0.05; 95% CI, 0.02-0.08) and beliefs that the pandemic would worsen (coefficient, 0.04; 95% CI, 0.01-0.06) were associated with lower willingness to receive vaccination. There was little evidence that willingness to receive vaccination varied with religion, as indicators for self-described nonevangelical Christians, evangelical Christians, and nonreligious study participants were all statistically insignificant (other religious affiliations comprising the baseline category). Wald tests also showed that none of the religion coefficients were significantly different from one another.

## Discussion

To our knowledge, this survey study provides the first systematic evidence of factors associated with individual preferences toward COVID-19 vaccination. Consistent with previous research, vaccine efficacy was associated with vaccine choice and likelihood of self-reported willingness to receive a vaccine.^12^ Also, similar to prior research on other vaccines,^16,17,18,19^ severe vaccine adverse effects were more likely to be associated with vaccine choice than mild side effects. Research on human coronaviruses suggests that lifetime immunity is unlikely.^20^ However, longer duration of immunity of 5 years vs 1 year was associated with higher support for vaccine choice, consistent with studies showing that duration of immunity was associated with individual vaccination preferences.^17,18^

As of April 30, 2020, the US FDA had issued EUAs for more than 170 medical products related to the COVID-19 pandemic; however, and consistent with past research on other vaccines,^21,22^ in this study, EUA approval for a vaccine was associated with participant hesitancy toward vaccination. Previous research has focused on the relative influence of family and friends, local physicians, and public health entities on individual willingness to vaccinate.^12,23,32^ Factors associated with recommendations and advice regarding a vaccine may be more complicated in the context of COVID-19 because of the unprecedented politicization of potential treatments and public health responses to the pandemic, as exemplified by the political and medical disputes over President Trump’s promotion of an untested treatment, hydroxychloroquine,^33^ and the administration’s decision to withdraw US funding from the World Health Organization in response to its alleged cover-up and mismanagement of the outbreak.^34^ The present analysis incorporated a range of endorsements to assess how trust in different political leaders and institutions was associated with the likelihood of willingness to receive a vaccine, which may be relevant for understanding vaccine hesitancy.^35^ Both Centers for Disease Control and Prevention and World Health Organization endorsements were associated with more likelihood of vaccination than political endorsements, suggesting that public outreach campaigns encouraging vaccination should rely on advice from health experts, perhaps to the exclusion of specific political figures.

These analyses also offer insights about the groups or characteristics that are likely to be associated with vaccine hesitancy, which can inform public health efforts to communicate effectively about the COVID-19 vaccine. Older people and, consistent with past research on the uptake of influenza vaccines,^36,37^ Black individuals and women reported being less likely, on average, to receive a vaccine against COVID-19. Accordingly, public health authorities might consider outreach strategies that address the specific concerns of older adults and minority communities that have been more susceptible to COVID-19.

### Limitations

This study has several limitations. First, the study participants represented a convenience sample of participants; however, the survey platform incorporated quota samples that approximate nationally representative samples in terms of demographic characteristics (eTable 1 in the Supplement).^29^ Nonetheless, future research might consider probability-based sampling to replicate the results. Second, even though conjoint analysis enabled evaluation of more attributes and combinations of attributes than in a full factorial design, attributes were economized based on evidence emerging from clinical trials of leading vaccine candidates or on the politics of vaccination. Evidence about the attributes of promising vaccines should lead to updated studies about vaccine attributes that had not yet emerged at the time of this study, such as the possibility that 2 vaccine doses rather than 1 vaccine dose may be required and evidence from phase III trials about the prevalence of major and minor adverse effects. Third, the information provided to study participants was based on hypothetical vaccines; the self-reported responses about vaccine choice and willingness to receive a hypothetical vaccine may not reflect vaccine choices and behaviors related to vaccine acceptance or hesitancy involving actual COVID-19 vaccines.

## Conclusions

In this survey study of US adults, vaccine-related attributes and political characteristics were associated with self-reported preferences for choosing a hypothetical COVID-19 vaccine and self-reported willingness to receive vaccination. These results may help inform public health campaigns to address vaccine hesitancy when a COVID-19 vaccine becomes available.

## Notes

Supplement. **eAppendix****.** Additional Information **eTable 1.** Comparative Sample Demographics **eTable 2.** Marginal Means for All Attribute Levels **eTable 3.** Alternate Operationalizations of Individual Vaccine Evaluations **eFigure 1.** Sample Choice Set **eFigure 2.** Effect of Vaccine Attributes on Vaccination Preferences
