# Error in Results

**DOI:** 10.1001/jamanetworkopen.2020.30649

**Published:** 2020-11-23

In the Original Investigation titled “Factors Associated With US Adults’ Likelihood of Accepting COVID-19 Vaccination,”^1^ published online October 20, 2020, in the last paragraph of the Results, the term “lower willingness” was incorrectly used in the third-to-last sentence of the paragraph; the correct term is “greater willingness.” This article has been corrected.
