# Supplementary material for: Factors Associated With US Adults’ Likelihood of Accepting COVID-19 Vaccination
Source: JAMA Netw Open. 2020 Oct 20;3(10):e2025594. doi: 10.1001/jamanetworkopen.2020.25594 (PMC7576409; doi:10.1001/jamanetworkopen.2020.25594)
Supplement: Supplement. — eAppendix. Additional Information eTable 1. Comparative Sample Demographics eTable 2. Marginal Means for All Attribute Levels eTable 3. Alternate Operationalizations of Individual Vaccine Evaluations eFigure 1. Sample Choice Set eFigure 2. Effect of Vaccine Attributes on Vaccination Preferences [file jamanetwopen-e2025594-s001.pdf]

Supplemental Online Content

Kreps S, Prasad S, Brownstein JS, et al. Factors associated with US adults' likelihood of accepting COVID-19 vaccination. *JAMA Netw Open*. 2020;3(10):e2025594. doi:10.1001/jamanetworkopen.2020.25594

eAppendix. Additional Information

eTable 1. Comparative Sample Demographics

eTable 2. Marginal Means for All Attribute Levels

eTable 3. Alternate Operationalizations of Individual Vaccine Evaluations

eFigure 1. Sample Choice Set

eFigure 2. Effect of Vaccine Attributes on Vaccination Preferences

This supplemental material has been provided by the authors to give readers additional information about their work.

© 2020 Kreps S et al. *JAMA Network Open.*

eAppendix. Additional Information

Sample Demographics

eTable 1 presents the demographics of our Lucid sample (n = 1,971) and compares its demographic composition to that of two other major social science surveys, the most recent versions of the American National Election Study and General Social Survey, as well as to demographics from the US Census American Community Survey.

Sample Choice Set

All subjects were asked to evaluate five pairs of vaccines. A sample choice set is presented in eFigure 1. The levels of each attribute for each vaccine were randomly assigned, and the order in which the attributes appeared in the choice set was randomized across survey respondents.

Additional Analyses

The experiment employed two approaches to measure the associations between vaccine characteristics and public willingness to vaccinate. Each subject completed five choice tasks. In each choice task, subjects evaluated two hypothetical COVID-19 vaccines with randomly assigned attributes as summarized in Table 1 of the text. After reading about each vaccine, subjects were first asked to choose whether they would get vaccine A or vaccine B, or whether they would choose not to get either vaccine. After making a discrete choice between the two vaccines, subjects were also asked to indicate how likely or unlikely they would be to get each vaccine individually on a 7-point Likert scale from "extremely unlikely" to "extremely likely." These questions afford a different test of the associations between each attribute and subjects' willingness to receive vaccination. From these questions, we created a dichotomous dependent variable coded 1 if the subject was "slightly," "moderately," or "extremely" likely to receive each hypothetical vaccine profile and 0 if not.

In the text, we employ two different analytical strategies. For the discrete choice question, we estimated a benchmark OLS regression model and plotted average marginal component effect sizes (AMCEs) in the top panel of Figure 1. For the second dependent variable measuring subjects' willingness to receive each hypothetical vaccine individually, we also estimated a benchmark OLS regression model and presented marginal means in the bottom panel of Figure 1. The two measures are closely related. In a fully randomized survey-experimental design, such as the one employed, AMCEs are the differences between marginal means of a given attribute-level and that attribute's baseline level, all else equal. Each approach has advantages for substantive interpretation. AMCEs estimate the association between each attribute-level and vaccination preferences compared to those at the attribute's baseline, whereas marginal means offer information about vaccination likelihood at all feature levels.

To complement the analyses presented in Figure 1 in the text, eFigure 2 presents the marginal means for the discrete choice question (Model 1 of Table 3) and the AMCEs for the individual vaccine likelihood dependent variable (Model 3 of Table 3). The results are substantively similar to those presented in Figure 1. The marginal means are lower in the discrete choice question because subjects could choose between three options here: vaccine A, vaccine B, or neither. However, the pattern of estimated effects is very similar. The AMCEs for the model of responses to the individual vaccine evaluation question are the differences in marginal means of each attribute-level from its baseline. Marginal means for all attribute-levels in both models are presented in eTable 2.

Finally, as robustness checks we re-estimated the analysis of the individual vaccine evaluation (Table 3, Model 3) using different operationalizations of the dependent variable. Model 1 of eTable 3 again uses a dependent variable coded 1 if the subject was "slightly," "moderately," or "extremely" likely to take each hypothetical vaccine profile and 0 if not. This model is identical to that reported in the text. Model 2 of eTable 3 uses a dependent variable coded 1 if the subject was only "moderately" or "extremely" likely to take the presented vaccine and 0 if not. Model 3 analyzes *unwillingness* to vaccinate, and uses a dependent variable coded 1 for subjects who report being

© 2020 Kreps S et al. *JAMA Network Open.*

"extremely," "moderately," or "slightly" unlikely to take the presented vaccine, and 0 if not. Finally, Model 4 uses an ordinal dependent variable reporting the full 7-point Likert scale. The results are substantively very similar across operationalizations of the dependent variable. The most important exception is that the coefficient for minor side effects is only statistically significant (i.e. $P<.05$, two-tailed test) in two of the four models. Protection duration and the Biden endorsement coefficients are significant in three of four models; the coefficients for all other vaccine attributes are statistically significant and in the expected direction for all four models.

Other Factors Associated with Vaccine Acceptance

Models 2 and 4 of Table 3 include a range of additional variables to examine the relationships between vaccine acceptance and subjects' health background and preferences; personal exposure to those affected by COVID-19; beliefs about the future course of the pandemic; political partisanship; and demographics, including gender, age, educational attainment, religious affiliations, and race.

We measure partisanship with two indicator variables identifying subjects who self-identify as a Democrat or a Republican; both measures include those who lean toward one party or the other. Educational attainment is measured on an 8-point scale from less than high school (2% of our sample) to professional degree (4%). Past frequency of flu vaccination is measured on a 4-point scale: never; once or twice; most years; every year. Favorability toward the pharmaceutical industry was measured on a 5-point scale from "very negative" to "very positive." Religious affiliation was measured with three dummy variables: non-Evangelical Christians (defined, following Pew, as Catholics, Protestants, Orthodox Christians, and Mormons who did not self-identify as Evangelical in a follow-up question); Evangelical Christians; and those who identified as atheists, agnostics, or "nothing in particular. The omitted baseline category captures subjects who identified as Jewish, Muslim., Buddhist, or Hindu (just over 10% of our sample, combined). Finally, the models included indicator variables for those identifying as Black or Latino.

© 2020 Kreps S et al. *JAMA Network Open.*

eTable 1: Comparative Sample Demographics

	Lucid Sample	2016 ANES	2018 GSS	US Census
Demographics				
Black	14%	9%	16%	13%
Latino	10%	11%	6%	18%
Female	51%	52%	55%	51%
% College degree	48%	39%	33%	32%
Median age	43 years	49 years	48 years	38 years
Political Characteristics				
Republican	34%	29%	23%	
Democrat	37%	34%	32%	
Ideology (% moderates)	33%	21%	38%	

Note: All Census figures taken from the 2018 American Community Survey. Partisan measures do not include those who lean toward one party or the other.

© 2020 Kreps S et al. *JAMA Network Open.*

eTable 2: Marginal Means for All Attribute Levels

	Discrete Choice			Individual Vaccine Evaluation		
	Mean	95% CI		Mean	95% CI	
Efficacy: 50%	0.32	(0.30	0.33)	0.51	(0.50	0.53)
Efficacy: 70%	0.39	(0.38	0.40)	0.56	(0.54	0.58)
Efficacy: 90%	0.48	(0.47	0.49)	0.61	(0.59	0.62)
Duration: 1 year	0.37	(0.36	0.38)	0.55	(0.54	0.57)
Duration: 5 years	0.42	(0.41	0.43)	0.57	(0.55	0.59)
Major: 1 in 10,000	0.36	(0.35	0.37)	0.54	(0.52	0.56)
Major: 1 in 1,000,000	0.43	(0.42	0.44)	0.58	(0.56	0.60)
Minor: 1 in 10	0.39	(0.38	0.40)	0.55	(0.53	0.57)
Minor: 1 in 30	0.40	(0.39	0.41)	0.57	(0.55	0.59)
FDA: Full approval	0.41	(0.40	0.42)	0.57	(0.56	0.59)
FDA: EUA	0.38	(0.37	0.39)	0.55	(0.53	0.57)
Origin: USA	0.45	(0.44	0.46)	0.60	(0.59	0.62)
Origin: UK	0.41	(0.40	0.43)	0.58	(0.56	0.60)
Origin: China	0.32	(0.31	0.33)	0.50	(0.48	0.52)
Endorsed: Trump	0.35	(0.34	0.37)	0.52	(0.50	0.54)
Endorsed: Biden	0.37	(0.36	0.39)	0.55	(0.53	0.57)
Endorsed: CDC	0.44	(0.43	0.46)	0.59	(0.57	0.61)
Endorsed: WHO	0.41	(0.40	0.42)	0.58	(0.56	0.60)

Note: Marginal means obtained from models 1 and 3 of Table 3.

© 2020 Kreps S et al. *JAMA Network Open.*

eTable 3: Alternate Operationalizations of Individual Vaccine Evaluations

	(Model 1)			(Model 2)			(Model 3)			(Model 4)		
	Coef.	95% CI	P-value	Coef.	95% CI	P-value	Coef.	95% CI	P-value	Coef.	95% CI	P-value
Efficacy: 70%	0.05	0.03 - 0.06	P<.001†	0.06	0.04 - 0.07	P<.001†	-0.05	-0.06 - - 0.03	P<.001†	0.25	0.18 - 0.32	P<.001†
Efficacy: 90%	0.09	0.07 - 0.11	P<.001†	0.10	0.09 - 0.12	P<.001†	-0.07	-0.09 - - 0.06	P<.001†	0.43	0.35 - 0.50	P<.001†
Duration: 5 years	0.02	0.00 - 0.03	P=.01†	0.04	0.02 - 0.05	P<.001†	-0.01	-0.03 - 0.00	P=.07	0.11	0.05 - 0.17	P<.001†
Major: 1 in 1,000,000	0.04	0.03 - 0.05	P<.001†	0.04	0.03 - 0.06	P<.001†	-0.04	-0.05 - - 0.02	P<.001†	0.19	0.13 - 0.25	P<.001†
Minor: 1 in 30	0.02	0.01 - 0.03	P=.004†	0.01	-0.00 - 0.03	P=.06	-0.01	-0.02 - 0.00	P=.06	0.06	0.00 - 0.12	P<03†
FDA: EUA	-0.02	-0.04 - - 0.01	P=.003†	-0.02	-0.03 - - 0.00	P=.009†	0.02	0.01 - 0.03	P=.001†	-0.10	-0.16 - - 0.04	P=.001†
Origin: UK	-0.02	-0.04 - - 0.00	P=.01†	-0.02	-0.04 - - 0.00	P=.02†	0.02	0.01 - 0.04	P=.004†	-0.12	-0.19 - - 0.05	P=.001†
Origin: China	-0.10	-0.12 - - 0.08	P<.001†	-0.09	-0.11 - - 0.07	P<.001†	0.10	0.09 - 0.12	P<.001†	-0.52	-0.60 - - 0.44	P<.001†
Endorsed: Biden	0.02	-0.00 - 0.04	P=.06	0.03	0.01 - 0.05	P=.003†	-0.03	-0.05 - - 0.00	P=.02†	0.15	0.05 - 0.25	P=.003†
Endorsed: CDC	0.07	0.05 - 0.09	P<.001†	0.06	0.04 - 0.08	P<.001†	-0.08	-0.10 - - 0.06	P<.001†	0.37	0.28 - 0.46	P<.001†
Endorsed: WHO	0.05	0.03 - 0.08	P<.001†	0.05	0.03 - 0.07	P<.001†	-0.06	-0.08 - - 0.04	P<.001†	0.29	0.20 - 0.39	P<.001†
Constant	0.49	0.46 - 0.52	P<.001	0.31	0.29 - 0.34	P.<001	0.35	0.33 - 0.38	P<.001	4.14	4.01 - 4.26	P<.001
Observations	19,710			19,710		19,710	19,710		19,710	19,710		19,710

Note: Regression coefficients and 95% confidence intervals. Model 1 uses a binary dependent variable coded 1 for subjects who report being “slightly”, “moderately”, or “extremely” likely to take the vaccine. Model 2 uses a binary dependent variable coded 1 for subjects who report being “moderately” or “extremely” likely to take the vaccine. Model 3 – analyzing *unwillingness to vaccinate* -- uses a binary dependent variable coded 1 if the subject reported being “extremely”, “moderately”, or “slightly” unlikely to take the vaccine. Model 4 uses the full seven-point ordinal scale ranging from extremely unlikely to extremely likely to take the vaccine presented. The results are very similar across operationalizations of the dependent variable. P-values for coefficients indicated with an † are below the adjusted target P value (adjusting for $\alpha = 0.05$) calculated via the Benjamani-Hochberg correction controlling for the false discovery rate in multiple comparisons.

© 2020 Kreps S et al. *JAMA Network Open.*

eFigure 1: Sample Discrete Choice Set

As you may know, scientists around the world are working to develop a vaccine for COVID-19. Please consider the hypothetical vaccines described in the table below:

	**Vaccine A**	**Vaccine B**
**Risk of severe side effects (hospitalization or death)**	1 in 10,000	1 in 10,000
**Endorsed by**	Vice President Joe Biden	President Donald Trump
**Risk of mild side effects (flu-like symptoms)**	1 in 30	1 in 30
**Vaccine origin**	United States	China
**Development and testing procedure**	The vaccine has been approved and licensed by the US Food and Drug Administration.	The vaccine has been approved and licensed by the US Food and Drug Administration.
**Efficacy – protection against severe symptoms such as respiratory failure, admission to a hospital ICU, or death**	70%	90%
**Protection duration**	5 years	5 years

Questions: If you had to choose, would you choose to get Vaccine A or Vaccine B, or would you choose not to be vaccinated?

How likely or unlikely would you be to get **Vaccine A** described above (7-point scale)?

How likely or unlikely would you be to get **Vaccine B** described above (7-point scale)?

© 2020 Kreps S et al. *JAMA Network Open.*

eFigure 2: Effect of Vaccine Attributes on Vaccination Preferences

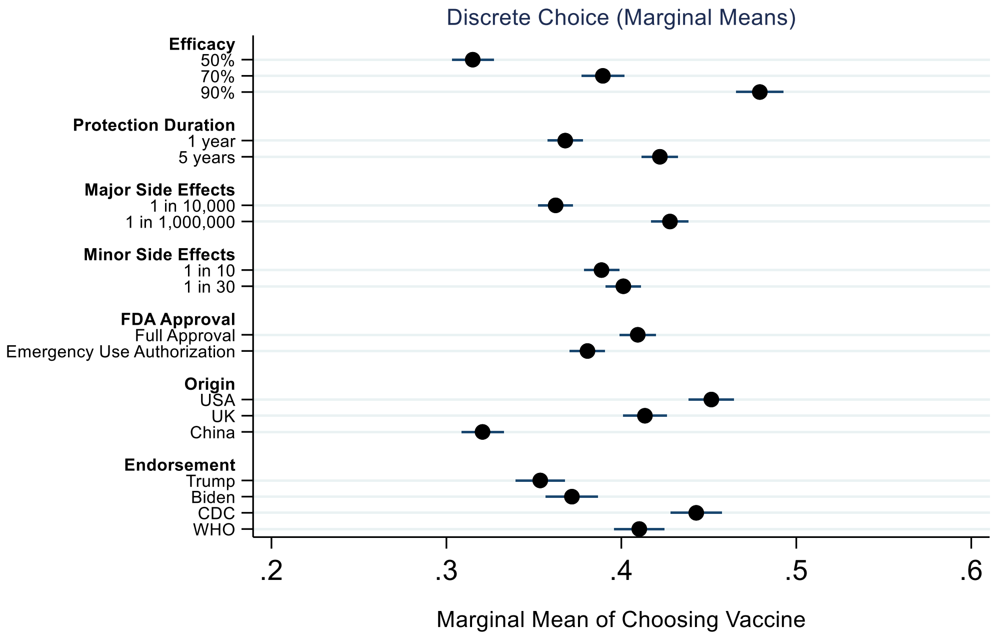

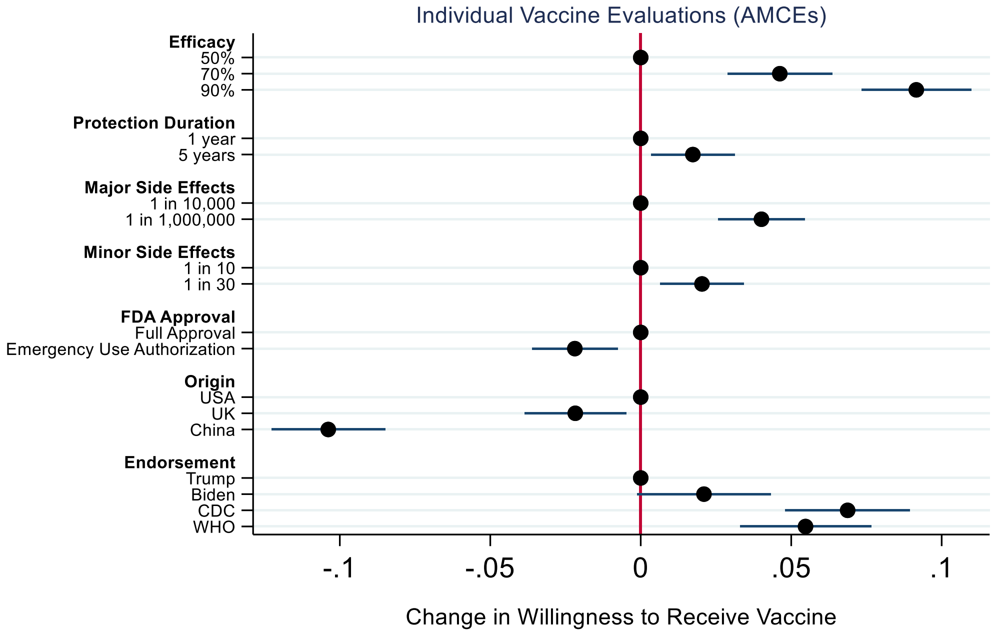

Note: The top panel shows the marginal means for each attribute value for choosing a hypothetical COVID-19 vaccine in the discrete choice setting. Estimates are based on Model 1 of Table 3. The bottom panel shows the average marginal component effect size for each attribute value for self-reported willingness to receive an individual hypothetical COVID-19 vaccine. The points without error bars denote the base level value for each attribute. Estimates are based on Model 3 of Table 3. In both panels, bars represent 95% confidence intervals around each point estimate.

© 2020 Kreps S et al. *JAMA Network Open.*
